# How Difficult Is Inference of Mammalian Causal Gene Regulatory Networks?

**DOI:** 10.1371/journal.pone.0111661

**Published:** 2014-11-04

**Authors:** Djordje Djordjevic, Andrian Yang, Armella Zadoorian, Kevin Rungrugeecharoen, Joshua W. K. Ho

**Affiliations:** 1 Victor Chang Cardiac Research Institute, Sydney, New South Wales, Australia; 2 The University of New South Wales, Sydney, New South Wales, Australia; Queen's University Belfast, United Kingdom

## Abstract

Gene regulatory networks (GRNs) play a central role in systems biology, especially in the study of mammalian organ development. One key question remains largely unanswered: Is it possible to infer mammalian causal GRNs using observable gene co-expression patterns alone? We assembled two mouse GRN datasets (embryonic tooth and heart) and matching microarray gene expression profiles to systematically investigate the difficulties of mammalian causal GRN inference. The GRNs were assembled based on 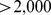 pieces of experimental genetic perturbation evidence from manually reading 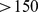 primary research articles. Each piece of perturbation evidence records the qualitative change of the expression of one gene following knock-down or over-expression of another gene. Our data have thorough annotation of tissue types and embryonic stages, as well as the type of regulation (activation, inhibition and no effect), which uniquely allows us to estimate both sensitivity and specificity of the inference of tissue specific causal GRN edges. Using these unprecedented datasets, we found that gene co-expression does not reliably distinguish true positive from false positive interactions, making inference of GRN in mammalian development very difficult. Nonetheless, if we have expression profiling data from genetic or molecular perturbation experiments, such as gene knock-out or signalling stimulation, it is possible to use the set of differentially expressed genes to recover causal regulatory relationships with good sensitivity and specificity. Our result supports the importance of using perturbation experimental data in causal network reconstruction. Furthermore, we showed that causal gene regulatory relationship can be highly cell type or developmental stage specific, suggesting the importance of employing expression profiles from homogeneous cell populations. This study provides essential datasets and empirical evidence to guide the development of new GRN inference methods for mammalian organ development.

## Introduction

Developmental biology, especially in the context of mammalian development, is the study of growth, differentiation, patterning and regeneration of cells, tissues and organs [1]. It is remarkable that all cells in a multicellular organism contain the same genome yet they express different sets of genes, and respond differently to the same genetic or signalling perturbation, in a highly regulated manner. It is increasingly clear that there is a need to fully unravel cell type-specific gene regulatory networks (GRNs) in order to understand the complex mechanisms underlying many developmental processes [2]–[4]. To achieve better understanding of complex genetic causes in organ development and diseases, we need to take a *systems approach* that interrogates causal genetic regulatory relationships (*e.g.*, conditional knockout of *Pax9* reduces the expression of *Msx1* [5]). Therefore in this study we mainly focus on the inference of *causal GRNs* in which each node represents a gene, and each edge represents a causal regulatory relationship between two genes.

The identification of causal gene regulatory relationships has a long history in the study of mammalian organ development, despite being primarily driven by hypothesis-based candidate gene investigations. Over the last half a century, developmental biologists have often used low throughput *in vivo* techniques such as *in situ* hybridization and immunohistochemistry to accurately detect spatio-temporal changes in gene expression in response to targeted gene knock-out, knock-down or over-expression experiments. By summarising the results of many of these experiments, we can incrementally infer reliable causal GRNs. A classic example of this is Eric Davidson's work on constructing and analysing GRNs in sea urchin and other animals [3], [4].

With the increasingly widespread availability of genome-wide expression profiling technologies, such as microarray and next-generation sequencing, we are now able to measure the expression levels of almost all the genes in the genome simultaneously. This gave rise to the tantalising prospect that we could infer GRNs from expression profiles [6], [7]. Although correlation between two genes does not imply causation, the converse is commonly implicitly assumed by many algorithms — that causal gene regulation leads to *observable* gene co-expression. This assumption implies that if one can properly remove non-causal edges from a network constructed on measures of gene co-expression, the remaining edges are likely causal [8], [9]. In other words, many people attempted to infer a GRN from gene co-expression data alone without explicitly making use of gene perturbation experimental data [10]. Even though the developers of these methods were likely aware of the underlying assumptions and limitation on interpreting a GRN inferred from gene co-expression data, it is quite possible that the end-users might treat each edge in the inferred GRN as having a causal regulatory role.

Large-scale community challenges, such as the Dialogue for Reverse Engineering Assessments and Methods (DREAM), have been conducted to evaluate GRN inference methods using *in silico* simulated data or a number of known GRNs in bacteria or yeast. Many approaches perform better than random when comparing to ‘gold standard’ perturbation experiments, although distinguishing true from false positives in even the most confident predictions from the best performing algorithms is infeasible given the total search space, usually several orders of magnitude larger [11]. Ongoing evaluations of the DREAM challenge have shown that although network inference is partially achievable in prokaryotic organisms, inference in eukaryotic organisms still remains a major challenge [12], [13].

We note that several methods have been designed to infer causal networks based on perturbation data and have been applied to study mammalian development. Wagner showed that theoretically, if there is no noise and missing value in the data, it is possible to infer a causal GRN of 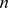 genes in 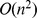 steps [14]. Nonetheless, real data contains noise and missing values. More sophisticated methods must be used. Nested effects models [15], [16] and methods based on deterministic effects propagation networks [17], [18] are effective at reconstructing the causal network between genes for which systematic (genome-wide) perturbation experiments exist. These algorithms are *not* the main focus of this study. We mainly focus on assessing the underlying assumption behind the algorithms that make use of gene co-expression data alone. Two such popular expression-based GRN inference algorithms are GENIE3 [19] and ARACNE [20].

A fundamental question arises, ‘Can we reverse engineer mammalian developmental causal GRNs from a collection of gene expression profiles?’. In addition, ‘How much can alternative data types contribute?’ To fully address this question, we will need high quality causal GRNs for comparison, but there is currently no gold standard for mammalian GRNs. Nonetheless, we have observed that there is a vast amount of experimentally validated genetic or molecular perturbation data in the published literature, but these data remain largely computationally inaccessible — mostly buried in figures, tables or text in developmental biology papers. Indeed it is exactly this type of data that is most often used as a gold standard for validation of predicted regulatory relationships and construction of high quality causal GRNs [11], [13], [21], [22].

## Methods

### Data summary

**Figure 1:**
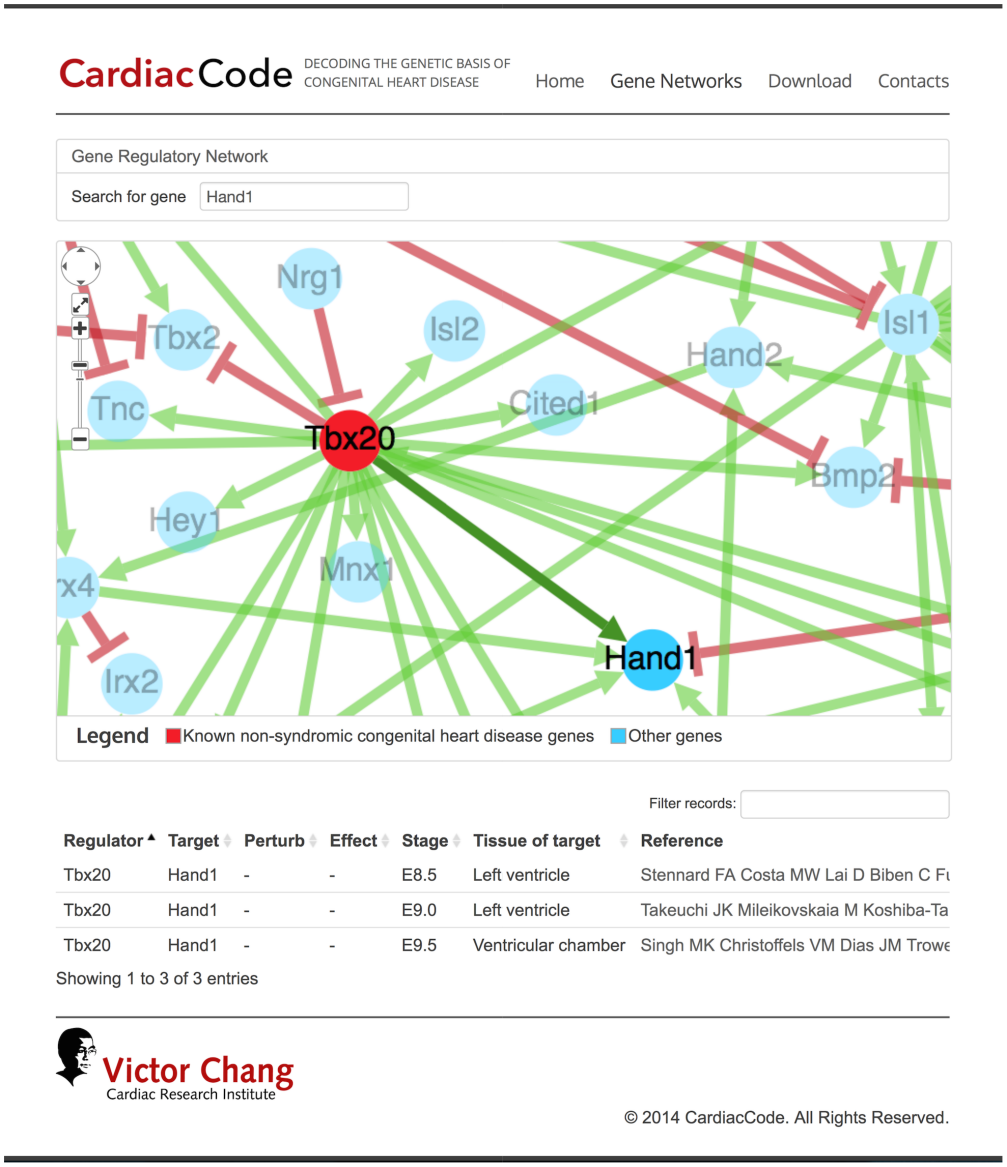
CardiacCode is a public online resource allowing interactive visualisation of the heart GRN, and download of the heart data collected and used in this study.

In this study, we assembled two manually-curated mouse GRN datasets (embryonic development of tooth and heart), summarising experimental evidence for causal regulation (or lack of causal regulation) between 1,177 pairs of regulator-target gene pairs, and a compendium of matching microarray expression profiles, to systematically investigate the difficulties of GRN inference in mammalian cells, especially in the context of organ development. The tooth GRN and microarray dataset was downloaded from ToothCODE, and the data were generated to study epithelial-mesenchymal interactions during early tooth organogenesis [23]. It contains over 1,500 pieces of genetic perturbation evidence from 120 primary research papers, and 105 matching microarray profiles (Table 1). Using a similar curation approach, we specifically assembled the heart dataset for this study. We manually collected over 700 pieces of genetic perturbation evidence from 43 published primary research papers on *in vivo* mouse cardiac development. We complemented this with 86 microarray expression profiles from the GEO database. The curated perturbation dataset, the assembled microarray data, and the inferred cardiac development network (see below for more details on inference of mode of regulation) can be accessed through our newly developed interactive web resource, CardiacCode (Figure 1). It was built on an SQL database and interfacing with javascript and HTML5 through PHP. The network visualisation was supported by the cytoscape.js plugin (Figure 1). The tooth and heart GRN and microarray gene expression datasets are available via ToothCODE (http://compbio.med.harvard.edu/ToothCODE/) and CardiacCode (http://CardiacCode.victorchang.edu.au/).

**Table 1:** Summary of tissue and time specific regulatory actions.

Data type	Feature	Tooth (ToothCODE)	Heart (this study)
Curated perturbation data	# Evidence	1518	710
	# Paper (years)	120 (1993–2011)	43 (1994–2013)
	# Regulators	109	32
	# Targets	160	129
	# 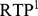	897	280
	# - Activating RTP	325	164
	# - Inhibiting RTP	117	36
	# - No effect RTP	455	80
Microarray data	Platform	Illumina	Affymetrix
	# Arrays	105	82
	# - Time series	45	40
	# - Genetic perturbation	24	30
	# - Signaling stimulation	36	0
	# - Phenotype difference	0	12

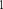
RTP: Regulator-Target Pair.

### Manual curation of genetic perturbation evidence from the literature

We recorded genetic perturbation experimental evidence from primary research papers. Each piece of evidence consists of 11 crucial pieces of information: regulator gene; target gene; perturbation performed on the regulator (+ or −); effect on the expression of the target gene (up-regulated, no change, down-regulated); species; developmental stage; tissue in which the perturbation was performed; tissue in which the expression of the target gene was measured; measurement technique; type of molecule measured (mRNA or protein); citation. If we were not confident about any of these pieces of information, the evidence was discarded. We further recorded the experimental context and additional information where it was available, including the genotype and phenotype of the perturbed mouse embryo.

### Inferring mode of regulation of a regulator-target pair

In this study, we only consider experimental evidence that comes from an *in vivo* embryonic mouse model (*i.e.*, not in cultured cells, or not in adult tissues), and was measured by *in situ* hybridisation, qRT-PCR, or similar well-established expression measurement techniques.

The regulator and target genes in each piece of experimental evidence form a regulator-target-pair (RTP). We define three possible *modes of regulations* for each RTP: activating, no interaction, and inhibiting. An edge is placed between two nodes in a GRN if its mode of regulation of the corresponding RTP is activating or inhibiting. We do not distinguish between direct and indirect interactions in this study, instead focusing solely on observable functional regulatory relationship between a regulator and a target gene. Since each RTP may be supported by multiple pieces of evidence, and they may not always be in total agreement, it is important to infer the mode of regulation of each RTP using a principled means.

First, we removed all RTPs that have opposite regulatory evidence in any tissues or time points — *i.e.*, observing both ‘activating’ and ‘inhibiting’. Afterwards, we used a probabilistic model to integrate the occasionally noisy data 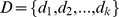 and estimate the mode of regulation 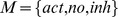 for each RTP. This method was first proposed by [23]. We specified a likelihood model 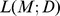 for each RTP,
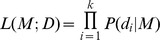



We then specified the likelihood model of observing each piece of evidence given the mode of regulation, 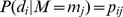, as a conditional probability matrix that describes the likelihood of observed experimental evidence for 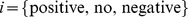 (rows of the matrix) given the true mode of interaction 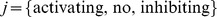 (columns of the matrix),
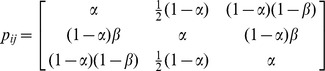
where 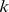 is the number of pieces of evidence corresponding to a RTP, 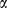 represents the probability of a correct experimental observation and 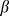 represents the probability of an incorrect experimental observation due to insensitivity of the detection technology. Here we used 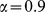 and 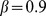, but our results are not sensitive to reasonable changes in these parameters [23].

The inferred mode of regulation is the mode 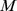 that maximises the likelihood function 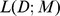.

### Microarray preprocessing

The tooth (Illumina MouseWG-6 v2.0) microarray gene expression data were downloaded from GEO (GSE32321) [23]. The heart (Affymetrix Mouse Genome 430 2.0) microarray data were assembled from multiple studies from GEO (Table S1). The assembled heart microarray profiles were quality checked, RMA normalized and log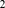 transformed (Figure S1, S2). In both datasets, low signal probes were removed (mean probe expression 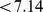 (Illumina) and 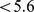 (Affymetrix) respectively). For differential gene expression analysis, we use the limma package [24] to determine statistically significantly up- or down-regulated genes (Benjamini-Hochberg adjusted 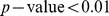). For inference of GRNs from microarray data, we use the 5000 most variable probes and all of the probes that matched regulator or target genes in our corresponding literature datasets were retained for further analysis.

### Network inference based on gene expression

#### Correlation

Correlation coefficients (Pearson and Spearman) were calculated on the subset of probes that matched the RTPs in the corresponding dataset. A representative correlation cut-off of 0.5 was used to define co-expression of the two genes represented by the two probes.

#### Mutual information

We use the *minet* R package [25] to calculate mutual information between the probes for all the RTPs.

#### ARACNE

We use the ARACNE algorithm [20], [26] as implemented in *minet*. Default settings were used, including ‘eps = 0’ for ARACNE to avoid prematurely throwing edges away.

#### GENIE3

The GENIE3 [19] algorithm was run using the R code provided by the authors. The random forest training step was parallelised using the *foreach* and *doParallel* libraries to improve efficiency on multi-core processors. In an attempt to standardise the network sizes between methods, the number of edges retrieved by the most unrestricted ARACNE adjacency matrix in each analysis was used to determine how many edges to retrieve from the GENIE3 weight matrix.

### Network inference based on other molecular networks

#### Protein-protein interactions

Protein-protein binding data was collected using the ‘iRefR’ R package [27]. Both human and mouse interaction data were used, resulting in a network of 448147 edges.

#### Pathway Commons data

Pathway information was downloaded from Pathway Commons (http://www.pathwaycommons.org/). Mouse and human specific edges were downloaded in.SIF format, giving 35088 and 392309 unique RTPs respectively.

### Calculating sensitivity and specificity of edge inference in GRNs

All of the networks, including those generated from the curated literature data and those inferred from other data sources, were encoded into graph structures using the ‘igraph’ R package [28]. Overlap of edges between two networks was calculated using the functions in the ‘igraph’ package. Area under the receiver operator characteristic curve was calculated using the ROCR R package [29].

## Results

### Causal gene regulation does not necessarily result in observable gene co-expression

**Figure 2:**
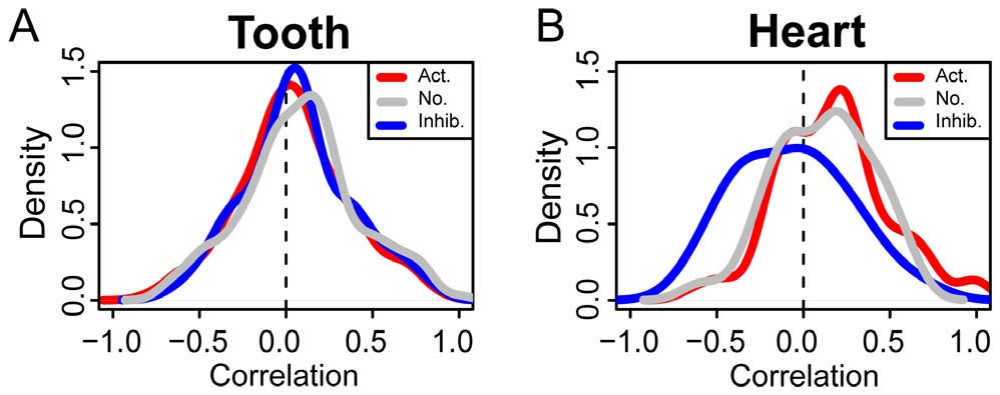
Spearman correlation of different classes of RTP in the tooth data (A) and the heart data (B). RTP classes are activating (Act.), no effect (No.) and inhibitory (Inhib.).

The assumption that a causal gene regulatory interaction should lead to an observable correlation of gene expression between the regulator and target is an attractive hypothesis that underlies many GRN reverse engineering approaches. If this assumption is true then we would expect certain trends, including an activating or inhibiting relationship having a positive or negative co-expression, respectively; and gene pairs that have been shown to have no regulatory relationship should have correlation coefficient close to zero. For each literature RTP, we calculated the Pearson and Spearman correlation, as well as the mutual information between the two genes across all of the matched microarray profiles (Figure 2).

In the tooth data, all RTPs, regardless of activating, inhibiting and no effect, have no or very weak Spearman correlation coefficients (Figure 2A). Neither do we observe a difference in Pearson correlation or mutual information values (Figure S3A, S4). This agrees with previous findings based on the *S. cerevisiae* GRN in the DREAM challenge [13]. In the heart data, there is a weak shift of the activating and inhibiting RTP towards higher and lower correlation values respectively (Figure 2B). Nonetheless, the gene co-expression patterns of the no-effect RTPs seem to be similar to that of the activating RTPs, suggesting in practice it would have been hard to distinguish true from false positive edges in a GRN if it was constructed based on gene co-expression.

### Common expression-based inference methods cannot reliably recover mammalian causal GRNs

**Figure 3:**
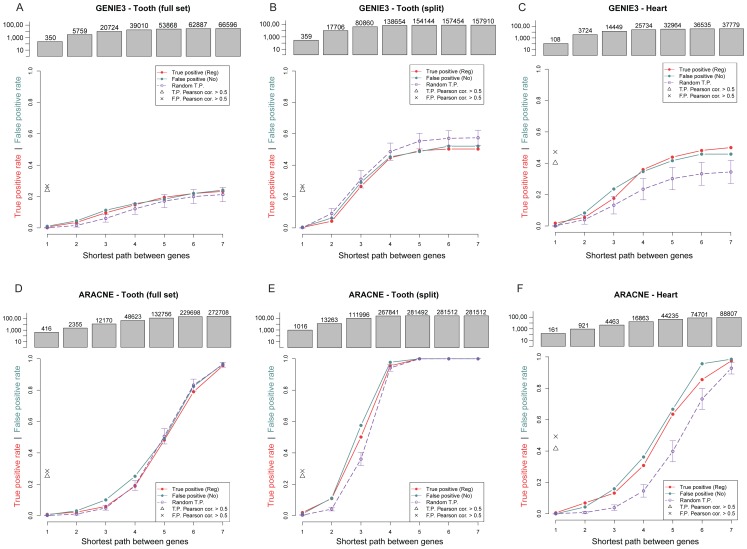
Evaluation of sensitivity (true positive rate) and specificity (1-false positive rate) of edge discovery by GENIE3 (A–C) and ARANCE (D–F) using the tooth and heart microarray datasets. To account for the possibility that our literature-curated RTP may represent indirect regulatory interactions, we allow matching of a RTP with a linear path of multiple edges (x-axis). The bar chart above each plot shows the size of the network. Dotted lines shows control background of 1,000 node-label-permuted randomised networks.

In order to investigate the usefulness of current GRN reverse engineering approaches applied to mammalian developmental gene expression data, we ran the GENIE3 and ARACNE algorithms on the tooth and heart microarray datasets and compared the resulting networks to our literature curated GRNs. These two algorithms were chosen because GENIE3 was shown to perform well in the recent DREAM challenge [13], and ARACNE was developed for inferring human GRNs. In general, we found that the algorithms did not offer a tangible improvement over random background in terms of detection sensitivity or specificity (Figure 3), *i.e.*, the area under the receiver operator characteristic curve (AUROC) is close to 0.5.

First we observed that at the first-neighbour level, neither algorithm returned more than one or two true positives on any dataset. Because ARACNE and GENIE3 both work by pruning supposedly indirect edges and we do not assume that our RTPs are all direct regulatory relationships, we also considered matching each literature-based RTPs with 2- to 7-edge paths. Although the true positive rate increased as expected, it was accompanied by an almost equivalent increase in the false positive rate. Furthermore, we found that the algorithms trained on the tooth dataset did not perform better than the randomly permuted networks of the same structure, and only performed slightly better than random in the heart dataset. This slight improvement might be due to the slightly stronger discriminatory gene co-expression signals between activating and inhibitory RTPs (Figure 2). Nonetheless, reliable GRN inference in both datasets is virtual impossible in practice based on these two algorithms.

We found that using an absolute Pearson correlation threshold of 0.5 identified 2871 unique RTPs from the heart data and 3528 unique RTPs from the tooth data once self loops have been removed. From these RTPs we could reproduce 24% of our activating and inhibitory edges from the tooth literature (true positives), however 26% of our no-effect edges (false positives) were also identified. In heart we observed a 42% true positive rate, coupled with a 49% false positive rate. The overall result is the same even if we use a different Pearson correlation cut-off, and the overall AUROC is close to 0.5 (Figure S7). The size of the inferred networks that must be analysed in order to retrieve the same true positive rate as Pearson correlation was often an order of magnitude larger than the correlation based networks. This indicates that in practice, interpretation of the results of GENIE3 and ARACNE may be more challenging, less beneficial and less intuitive than analysing a Pearson correlation based network, although neither will consistently return more true positives than false positives.

### Microarray perturbation results are consistent with the literature-curated RTPs

**Figure 4:**
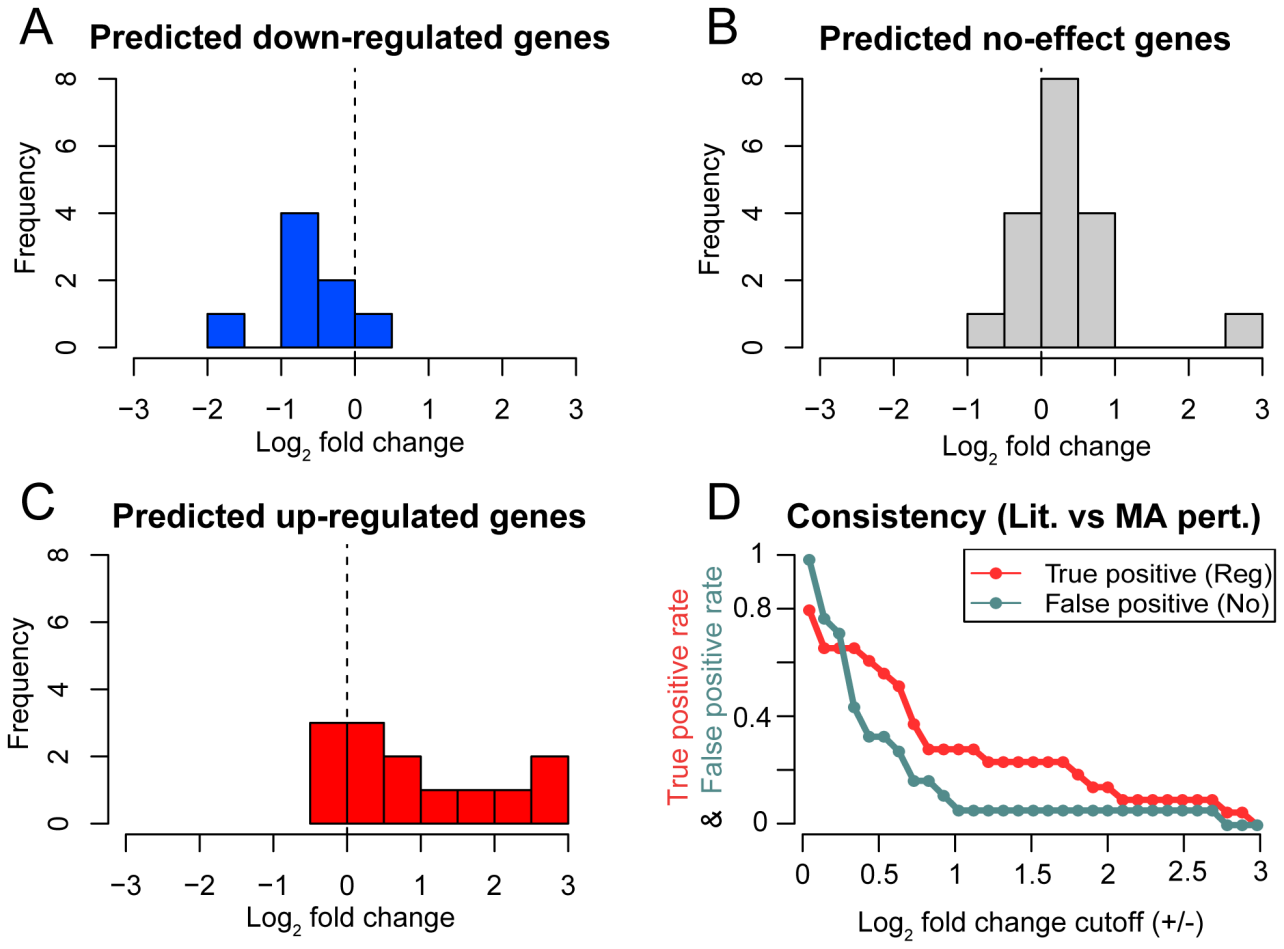
Fold changes (log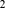) from tooth microarray perturbation experiments that matched the perturbation evidence in the literature show consistency with expected trends. RTPs that are inhibiting (A), have no effect (B), or are activating (C) trend to have negative, close to zero and positive fold changes respectively. (D) shows the consistency of the literature based RTP type (Lit.) and microarray data (M.A.) as fold change cut-off varies between 0 and 3 (both up- or down-regulation).

To examine whether the microarray data actually contain any information for identifying causal regulatory interactions, we investigated whether the set of differentially expressed genes from perturbation experiments can be used to infer causal regulatory relationships. The tooth microarray dataset contains 6 perturbation experiments, including transgenic knockdowns of *Msx1* and *Pax9*, and exogenous stimulation of the *BMP*, *Wnt*, *sonic hedgehog* and *FGF* pathways. Based on the pathway information provided by [23], we identified 39 RTPs from the literature at stage E13 that corresponded to the microarray perturbation experiments. Encouragingly, the observed directions and fold changes of differential expression as determined by the microarray experiments were consistent with the regulatory relationships predicted by the literature (Figure 4). We found that using a fairly conservative absolute log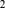 fold change cut-off of 1 (*i.e.*, 2-fold change) would result in a edge detection sensitivity (true positive rate) of 30%, increasing up to 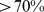 as the cut-off is relaxed. The false positive rate ( = 1-specificity) is consistently much lower than the true positive rate, suggesting that it is possible to distinguish causal gene regulation from non-regulatory ones with a reasonable sensitivity and specificity. We repeated the analysis considering all developmental stages, which increased the number of RTPs to 144. The trends are still visible although with increased noise (Figure S5).

### Tissue and temporal specificity is a confounding factor in network reconstruction

**Figure 5:**
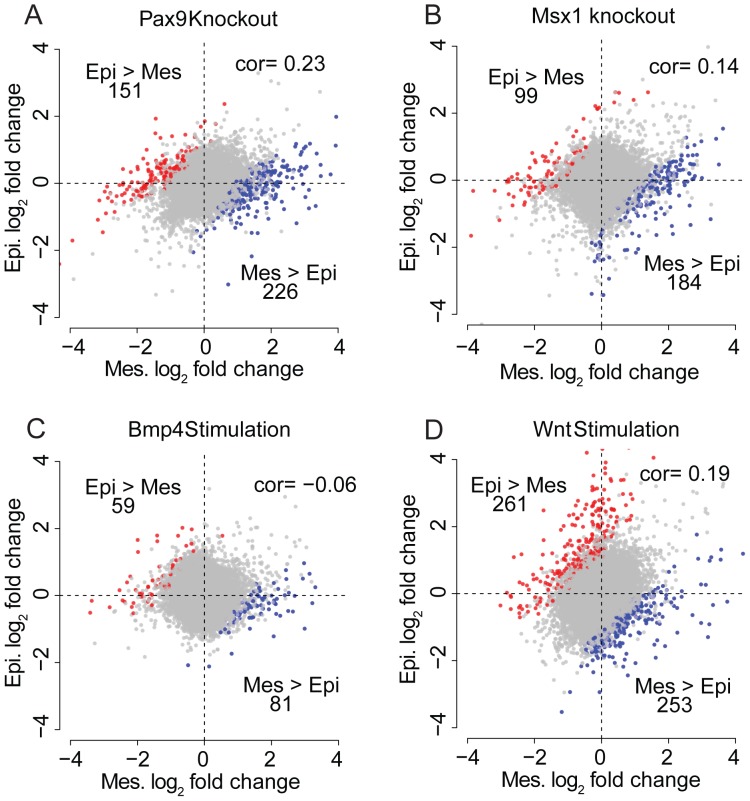
Scatter plots show the extent of tissue-specific differential expression in dental epithelium (y-axis) and dental mesenchyme (x-axis) as a result of *Pax9* knockout (A), *Msx1* knockout (B), *Bmp4* stimulation (C) and *Wnt* stimulation (D). Coloured points represent probes of differentially responsive genes between the two tissues. Pearson correlation is also shown.

We sought to investigate the extent to which different tissues display different genetic responses to the same stimulus. Using the ToothCODE microarray profiles on genetic perturbation experiments, we found that the magnitude of tissue specific responses varies considerably between different perturbations. First we examined epithelial and mesenchymal tissue microarray profiles from *Pax9* 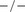 and *Msx1* 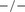 mice (Figure 5A,B). We identified hundreds of genes are significantly differentially expression in only one tissue type and not the other, even in the same genetic mouse model (FDR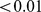). Similarly, we observed that distinct sets of genes are differentially expressed in response to the same signalling pathway stimulation (BMP and Wnt) in dental epithelium versus dental mesenchyme (Figure 5C,D; see also Figure S6). In addition, we also observed many tissue and/or temporal specific causal gene regulation in our tooth and heart literature datasets (Table S2). These results suggest that the causal gene regulatory network structure may be specific to individual cell or tissue types. Therefore, it is important to consider cell-type specificity when constructing GRNs in multicellular organisms [30], [31].

### The value of using perturbation data for GRN inference

It has been commonly believed that it is best to infer GRNs using expression profiles from a broad range of diverse conditions. To achieve such a diversity, we might collect samples from multiple cell types, multiple genetic perturbation, or developmental time series. To investigate the relative value of using perturbation vs. time series data, we split the heart microarray dataset into: 1) arrays from wildtype time-series experiments; 2) arrays from perturbation experiments. To see if the correlation shift trends arose we plotted the Pearson correlation for each type of RTP (Figure S3). We observed that the correlation of activating RTPs in the perturbation subset is generally higher than that observed in the time-series subset. The combined set seems to yield the best result.

We have shown that there can be a slight shift in the overall distribution of correlation values between activating and inhibiting causal relationships, but not to the extent where a cutoff can accurately differentiate these two classes from false positives (Figure 2, S3). How much information can be gained by exploiting perturbation experiments? We calculated the AUROC for Pearson correlation of all our activating or inhibiting RTPs compared to our no-effect RTPs, and similarly for the fold change values observed in matching perturbation microarray experiments (Figure S7). We clearly see that fold change from direct perturbation experiments is a much better predictor of causal gene regulation than Pearson correlation, with AUROCs of 0.63–0.87 compared to 0.55 based on gene co-expression alone.

### GRN inference based on protein interaction network and other molecular pathways

**Figure 6:**
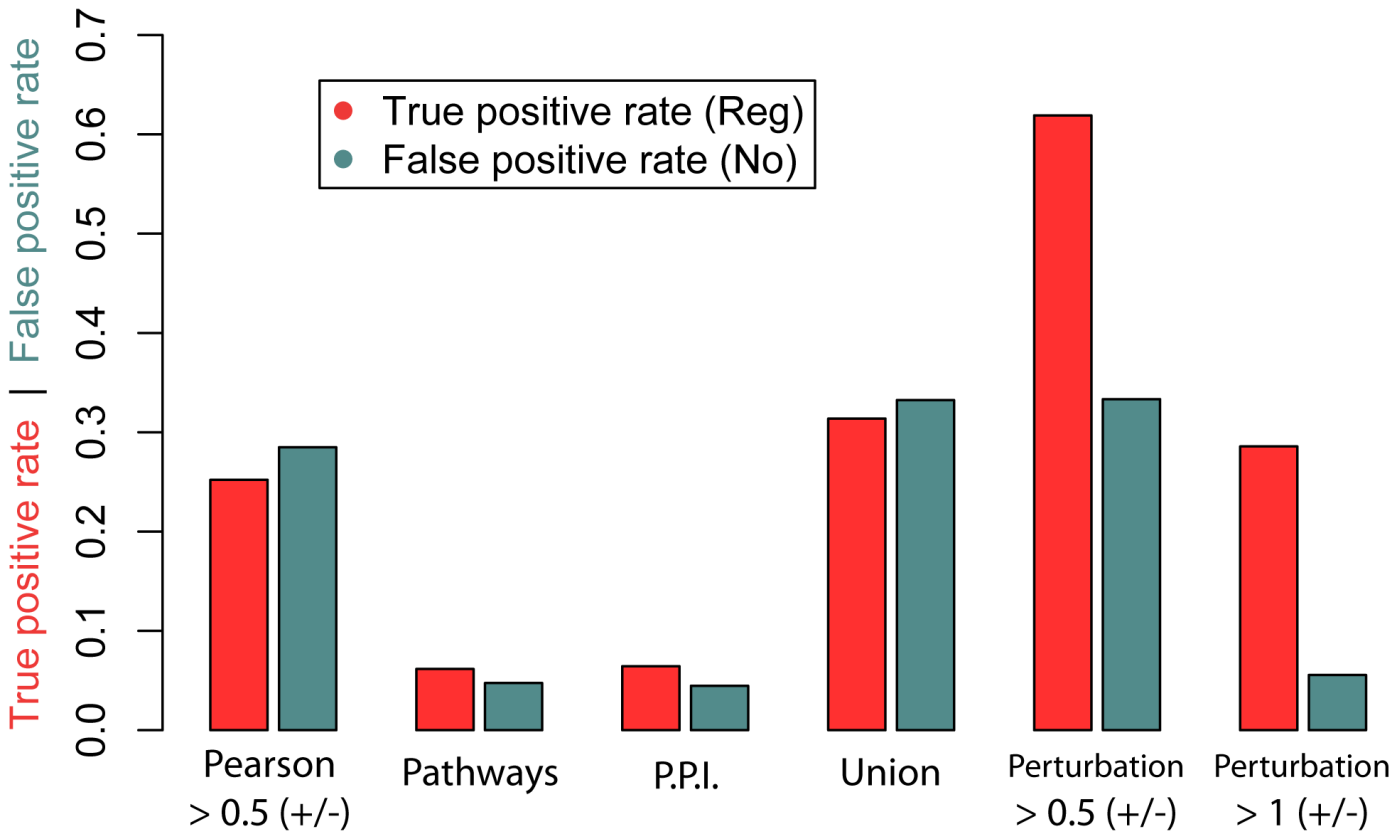
Comparison of the true positive and false positive rates as determined by different network inference approaches on the tooth dataset: Pearson correlation, Pathway Commons database, protein-protein interactions (PPI), the union of the previous three methods and direct effect on genetic perturbation (log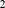 fold change cut-off or 0.5 and 1). Note: the TP and FP rates for the first 4 methods were calculated based on the subset of 686 RTPs that were represented in the microarray, PPI and pathway data. The TP and FP rates for perturbation data were based on the subset of 39 RTPs with a regulator matching the pathway being perturbed.

Using co-expression (as determined by Pearson correlation), we could achieve a true positive rate of 25%, but with almost a 30% false positive rate. We found that only 3–6% of the edges in Pathway Commons pathways or protein-protein interaction networks overlap with activating or inhibiting RTPs, however in all cases a similar proportion of false positives was also retrieved (Figure 6, Figure S8). By explicitly taking into account the perturbation design (as in Figure 4), we can significantly increase the true positive rate while keeping the false positive rate low (Figure 6, Figure S8).

## Discussion

This study aims to evaluate the practical utility of genome-wide expression profiles to infer causal gene regulatory networks in mammalian organ development. In particular, we assessed whether it is possible to observe gene co-expression in experimentally verified causal gene regulatory relationships — a common assumption in most GRN inference algorithms [7], [13]. One of the major results from the DREAM5 challenge is that many inference methods performed well when analysing *in silico* datasets and prokaryotic (*E. coli*) datasets, but inference of eukaryotic GRNs (in *S. cerevisiae*) is very poor regardless of which method was used [13]. Marbach et al. (2012) attributed the reduced inference accuracy to an increased regulatory complexity and prevalence of post-transcriptional regulation in eukaryotes. Ensemble-based approaches that combine multiple inference methods have been shown to slightly improve the inference accuracy [13].

We have gathered two well validated literature-curated datasets and matching microarray gene expression datasets to systematically evaluate the challenges of causal GRN inference. Our datasets are unique because they contain thorough annotation of tissue types and embryonic stages, as well as the type of regulation observed (activation, repression and no effect), which importantly allows us to estimate both sensitivity and specificity of inference of GRN edges. To our knowledge this study contains the most extensive evaluation of commonly applied GRN inference paradigms to mammalian embryogenesis and the first quantification of the difficulty of their application to this context. Our results show that inference of causal GRNs for mammalian developmental systems by considering gene co-expression alone is likely not an effective approach. Nonetheless, perhaps not too surprisingly, it is possible to infer causal regulatory relationships with good sensitivity and specificity if perturbation data are used. This result supports the importance of considering these data when reconstructing causal regulatory networks.

Our study place a strong emphasis on embryonic organ development. From a practical point of view, we chose this emphasis because of the wealth of data we have already collected (*e.g.*, the published ToothCODE data), the availability of a large amount of matching published microarray gene expression data from GEO, and the many reported successful applications of GRN to study developmental biology problems, such as Eric Davidsons work [1], [3], [4]. In this sense, the process of GRN inference should be easier than other non-developmental GRNs. From a conceptual point of view, the inference of developmental GRN is at least as difficult as, if not more difficult than, the inference of other GRNs since a useful developmental GRN will need to deal with regulatory relationships between multiple cell types, and the regulatory relationship between two genes may change dramatically during successive developmental stages. Therefore, we expect the lessons learned from our study will be informative to the inference of other non-developmental GRNs.

Our tooth and heart microarray datasets each have about 100 microarray samples, containing about 30 conditions. It is conceivable that better performance can be achieved by profiling more samples in additional conditions. Nonetheless, we noticed that it is practically not easy to obtain such data when studying*in vivo* gene expression patterns in embryonic animal models. Embryonic dissection, tissue collection and processing all require time, money and labour.

We did not extensively test the effect of microarray probe normalisation procedure. We simply follow common practices in microarray analysis, and ask whether this is quantitatively sufficient to recover information for GRN construction. It is conceivable that a more extensive enumeration of microarray processing procedure may yield higher correspondence with the literature network, but considering the high false positive rate observed in our current dataset, we do not expect the major results to change.

### Lessons for mammalian causal GRN inference

During the course of manually curating the literature data, we observed that there is a vast amount of genetic or molecular perturbation data in the published literature that largely remains computationally inaccessible. Unlike microarray or high throughput sequencing data, most people do not deposit the results of their perturbation results into a centralised database such as EBI ArrayExpress [32] or NCBI GEO [33]. Based on our experience, an undergraduate-level biology student can read 2–3 papers a day, and each paper contains on average 12 useful pieces of perturbation data. In one month, a single person can curate up to 700 pieces of perturbation data. Ultimately we would like to see a similar centralised repository where authors and researchers submit their own spatio-temporally annotated perturbation results at the time of publication, but there is currently no standard for reporting and annotating these dataset. Our experience on manual curation has been generally very positive and rewarding.

Considering the amount of gene perturbation data that one can obtain from simply computerising existing records, we believe this suggests that the community of computational systems biologist investigating mammalian disease and development should perhaps re-prioritise their research effort, *e.g.*, instead of focusing on inferring causal GRNs from high throughput genome-wide datasets, committing resources to systematic generation and curation of relevant genetic perturbation data, and developing algorithms to construct cell type and developmental stage specific GRNs from these potentially sparse and noisy perturbation data.

## Supporting Information

Figure S1 **Correlation matrix of cardiac microarray data downloaded from GEO.**
(TIFF)

Figure S2 **Boxplots showing RMA normalised cardiac microarray data downloaded from GEO.**
(TIFF)

Figure S3 **Pearson correlation kernel density plots for each class of RTP in heart, based on the complete microarray set (A), only the perturbation arrays (B) and only the time series (C).**
(TIFF)

Figure S4 **Mutual information kernel density plots for each class of RTP in heart (A) and tooth (B).**
(TIFF)

Figure S5 **Fold changes from tooth microarray perturbation experiments that matched the perturbation evidence in the literature (all stages) show consistency with expected trends.** Regulatory relationships that are inhibitory (A), have no effect (B), or are activating (C) trend to have negative, close to zero and positive fold changes respectively. (D) shows the consistency of the literature based predictionsand microarray data as fold change cutoff is increased.
(TIFF)

Figure S6 **Negative (blue) and positive (green) control distributions for analysing tissue-specific genetic responses to the same perturbation.** Positive control is generated by correlation of fold change of biological replicates. Negative control is correlation of independent experiments.
(TIFF)

Figure S7 **ROC curves showing the ability of perturbation experiments (A) and gene expression correlation (B) to differentiate regulatory from non-regulatory edges.**
(TIFF)

Figure S8 **Comparison of the true positive and false positive rates as determined by different network inference approaches on the heart data set: Pearson correlation threshold on 82 microarray profiles, Pathway Commons database, protein-protein interactions (P.P.I.), and the union of the previous three methods.**
(TIFF)

Table S1 **Summary of heart microarray dataset.**
(PDF)

Table S2 **Summary of the tooth and heart datasets.**
(PDF)
