# Supplementary figures and images for: How Difficult Is Inference of Mammalian Causal Gene Regulatory Networks?
Source: PLoS One. 2014 Nov 4;9(11):e111661. doi: 10.1371/journal.pone.0111661 (PMC4219746; doi:10.1371/journal.pone.0111661)

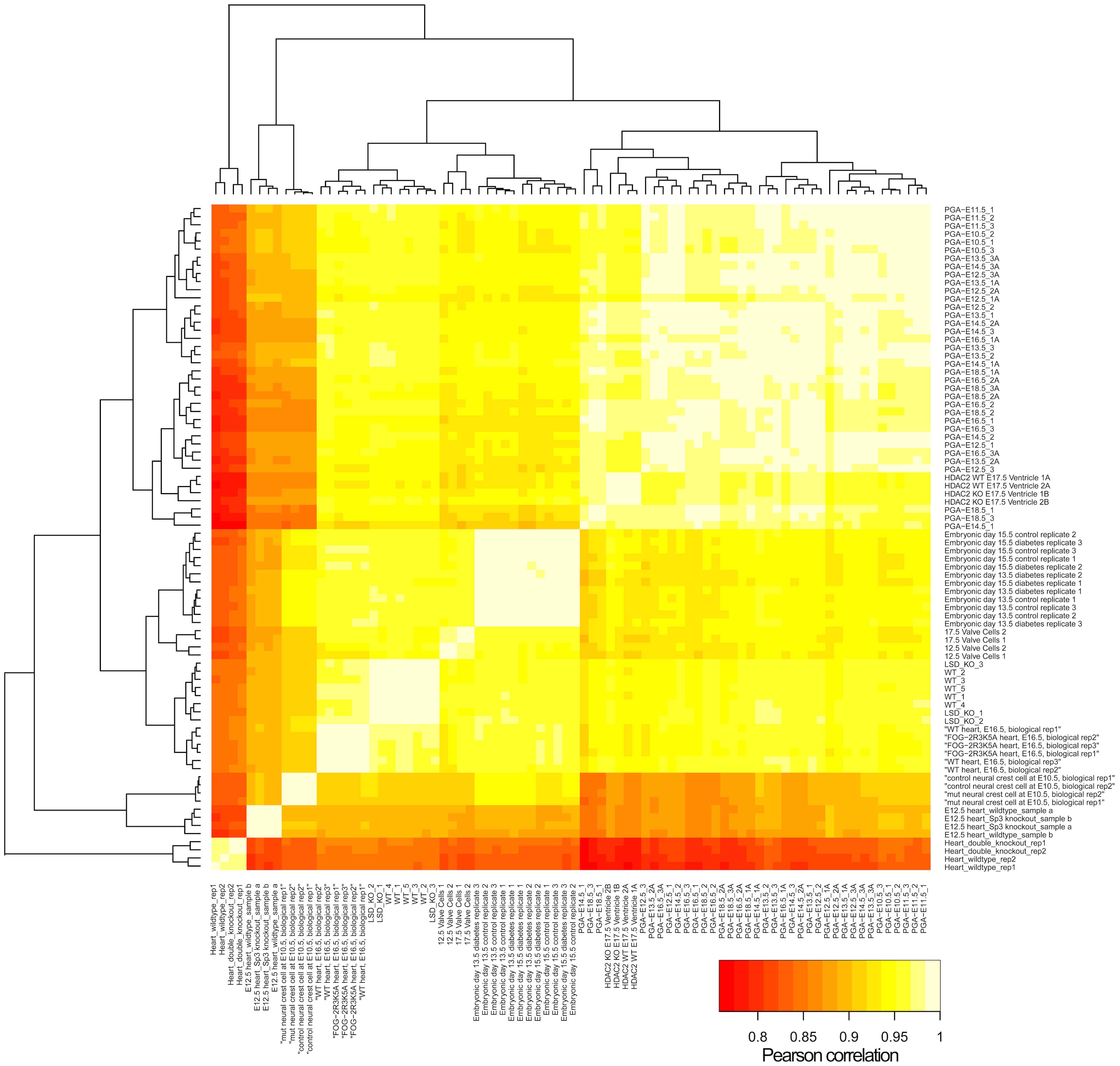

Supplement: Figure S1 — Correlation matrix of cardiac microarray data downloaded from GEO. (TIFF) [file pone.0111661.s001.tiff]

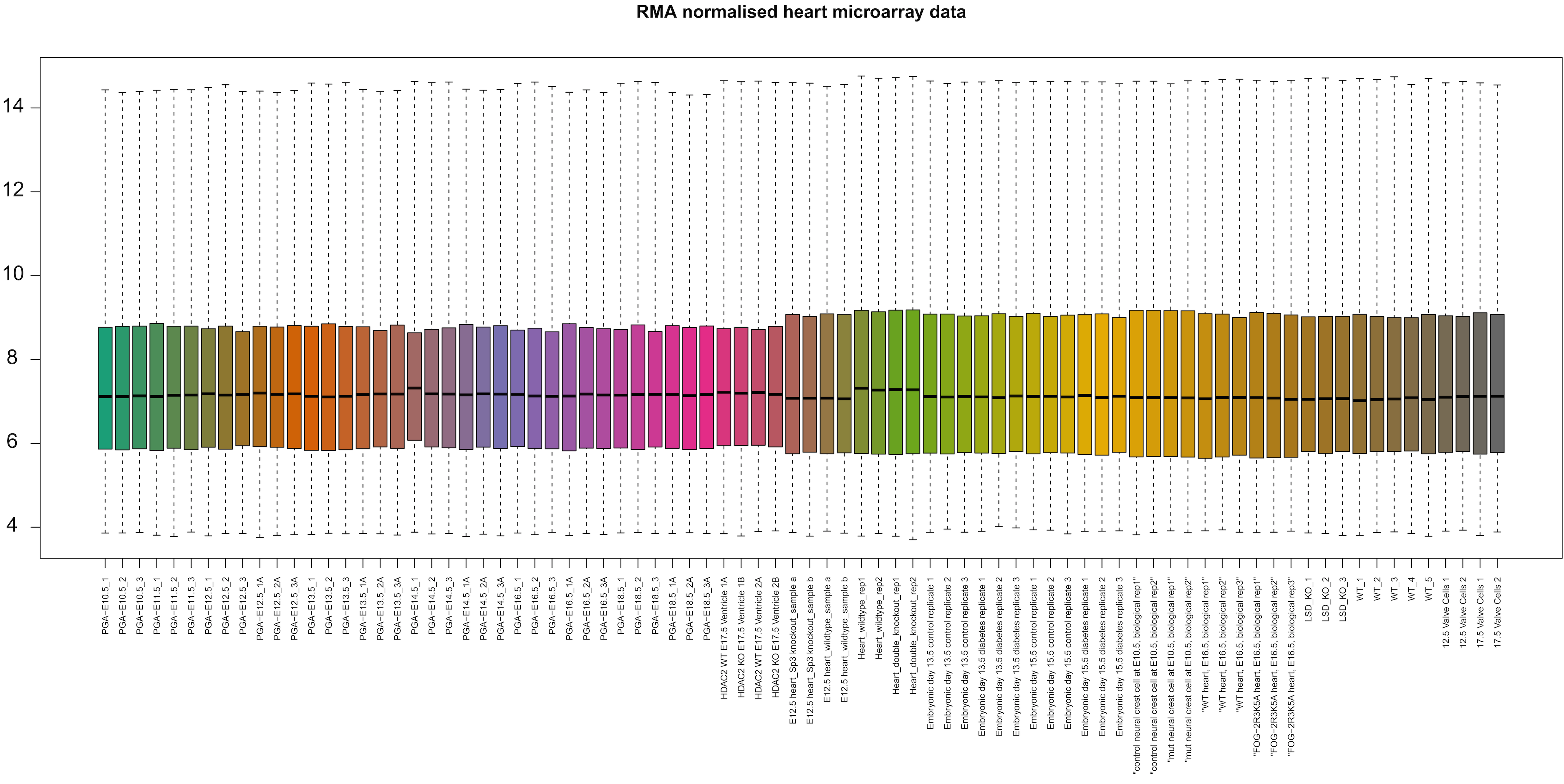

Supplement: Figure S2 — Boxplots showing RMA normalised cardiac microarray data downloaded from GEO. (TIFF) [file pone.0111661.s002.tiff]

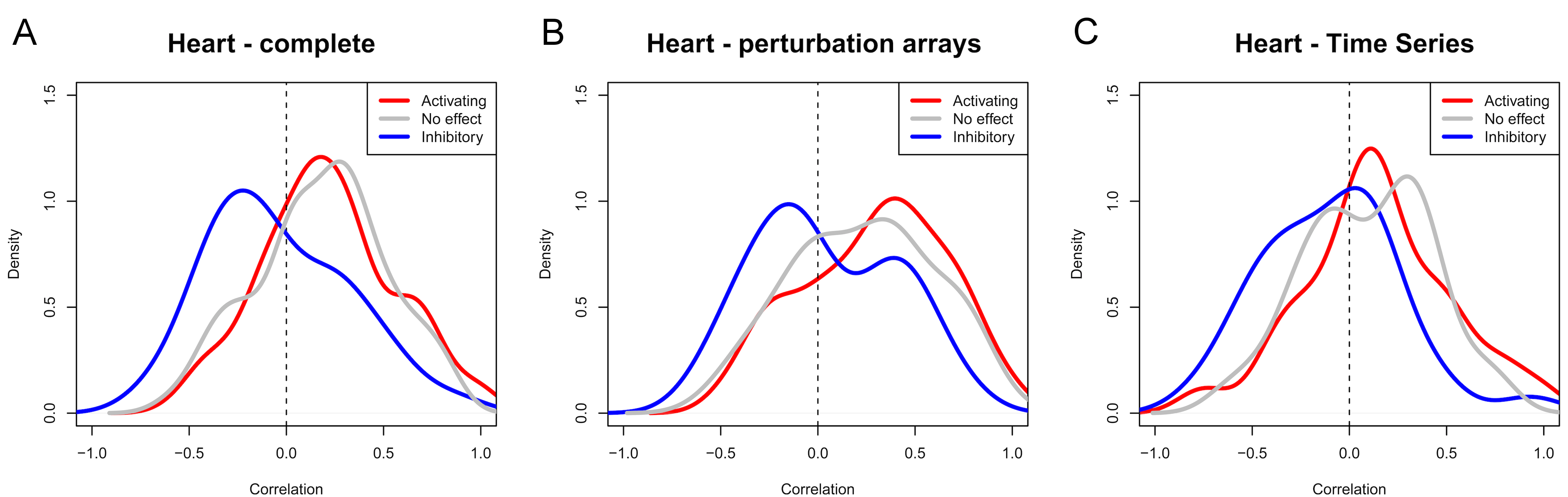

Supplement: Figure S3 — Pearson correlation kernel density plots for each class of RTP in heart, based on the complete microarray set (A), only the perturbation arrays (B) and only the time series (C). (TIFF) [file pone.0111661.s003.tiff]

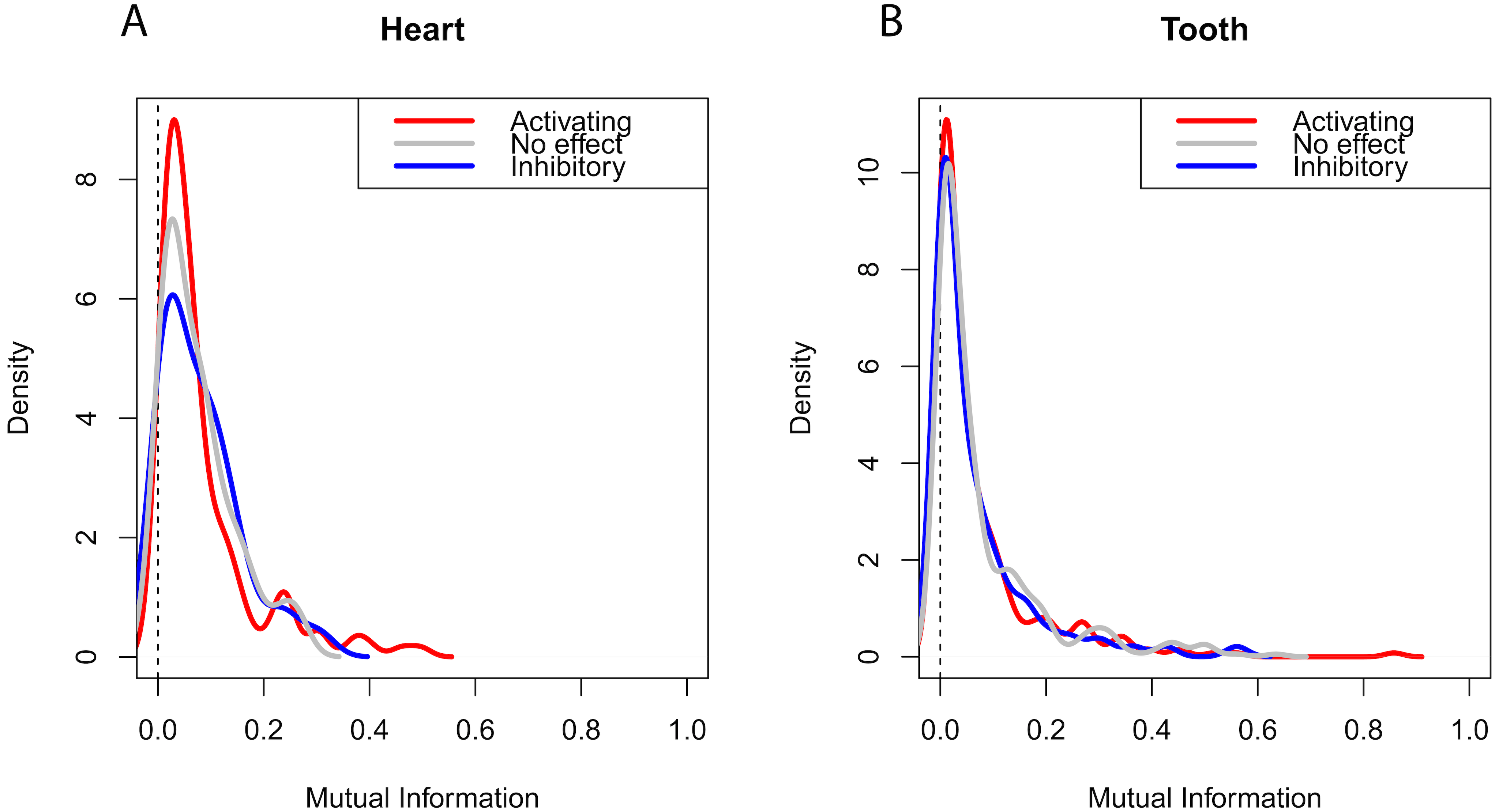

Supplement: Figure S4 — Mutual information kernel density plots for each class of RTP in heart (A) and tooth (B). (TIFF) [file pone.0111661.s004.tiff]

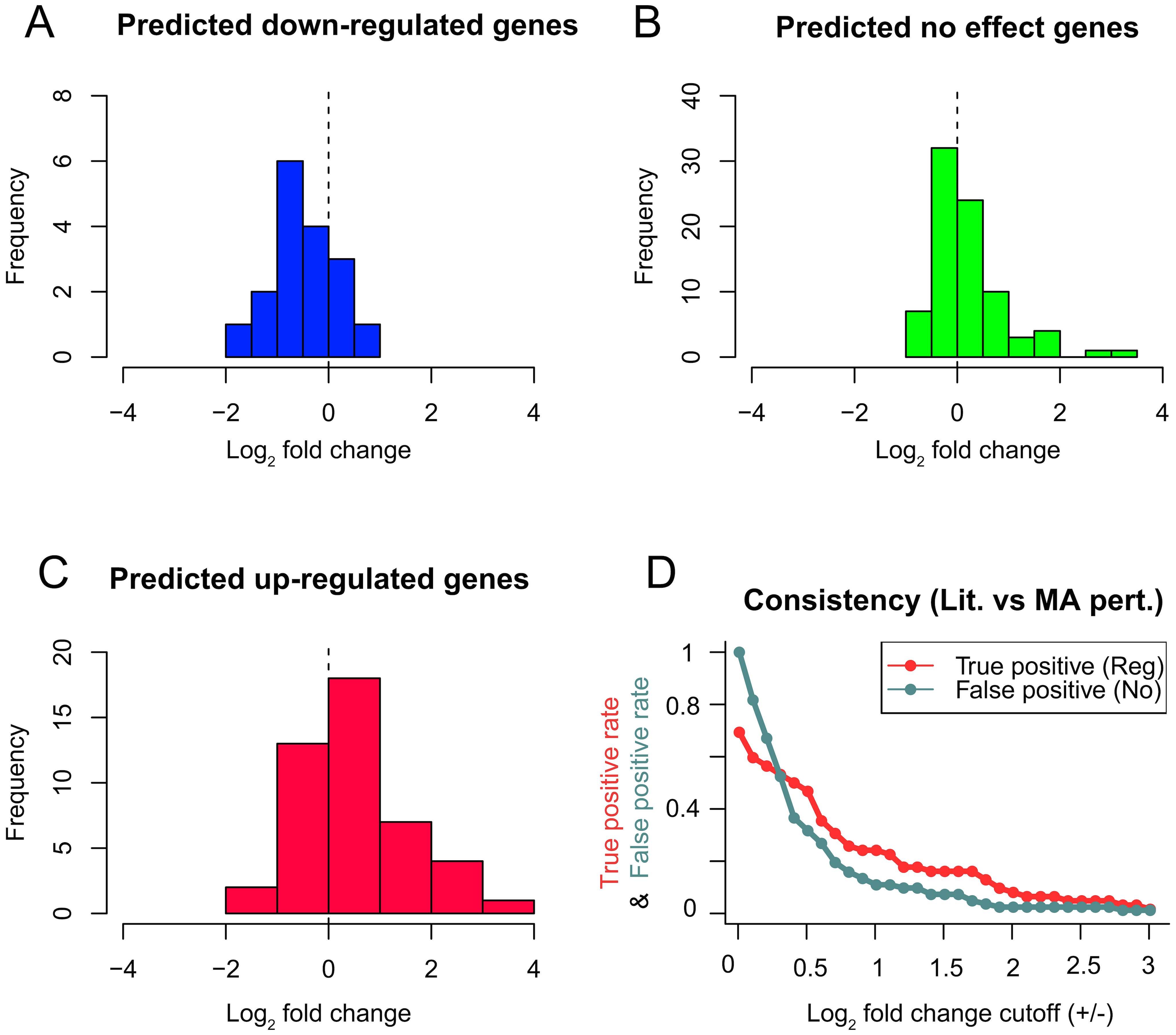

Supplement: Figure S5 — Fold changes from tooth microarray perturbation experiments that matched the perturbation evidence in the literature (all stages) show consistency with expected trends. Regulatory relationships that are inhibitory (A), have no effect (B), or are activating (C) trend to have negative, close to zero and positive fold changes respectively. (D) shows the consistency of the literature based predictionsand microarray data as fold change cutoff is increased. (TIFF) [file pone.0111661.s005.tiff]

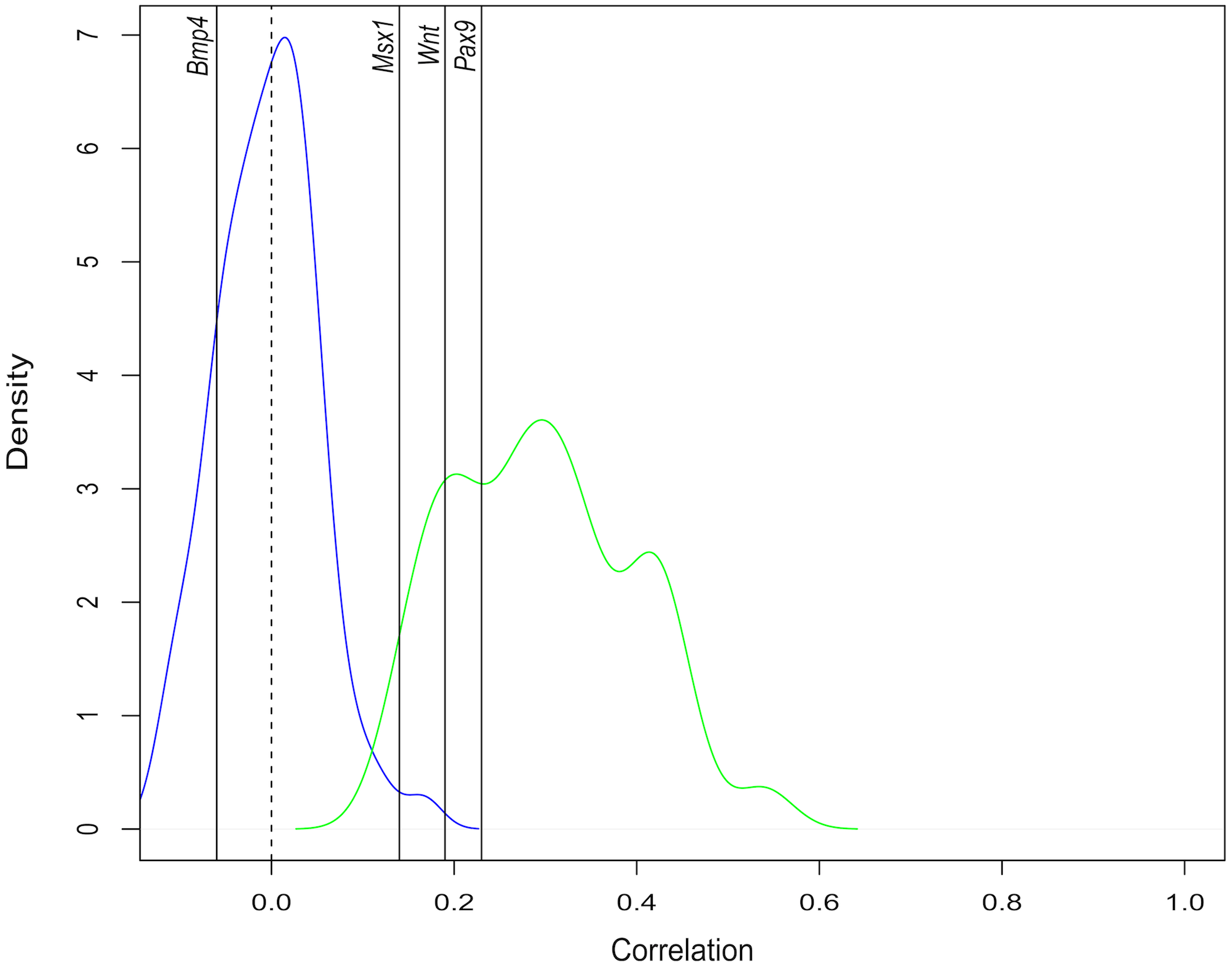

Supplement: Figure S6 — Negative (blue) and positive (green) control distributions for analysing tissue-specific genetic responses to the same perturbation. Positive control is generated by correlation of fold change of biological replicates. Negative control is correlation of independent experiments. (TIFF) [file pone.0111661.s006.tiff]

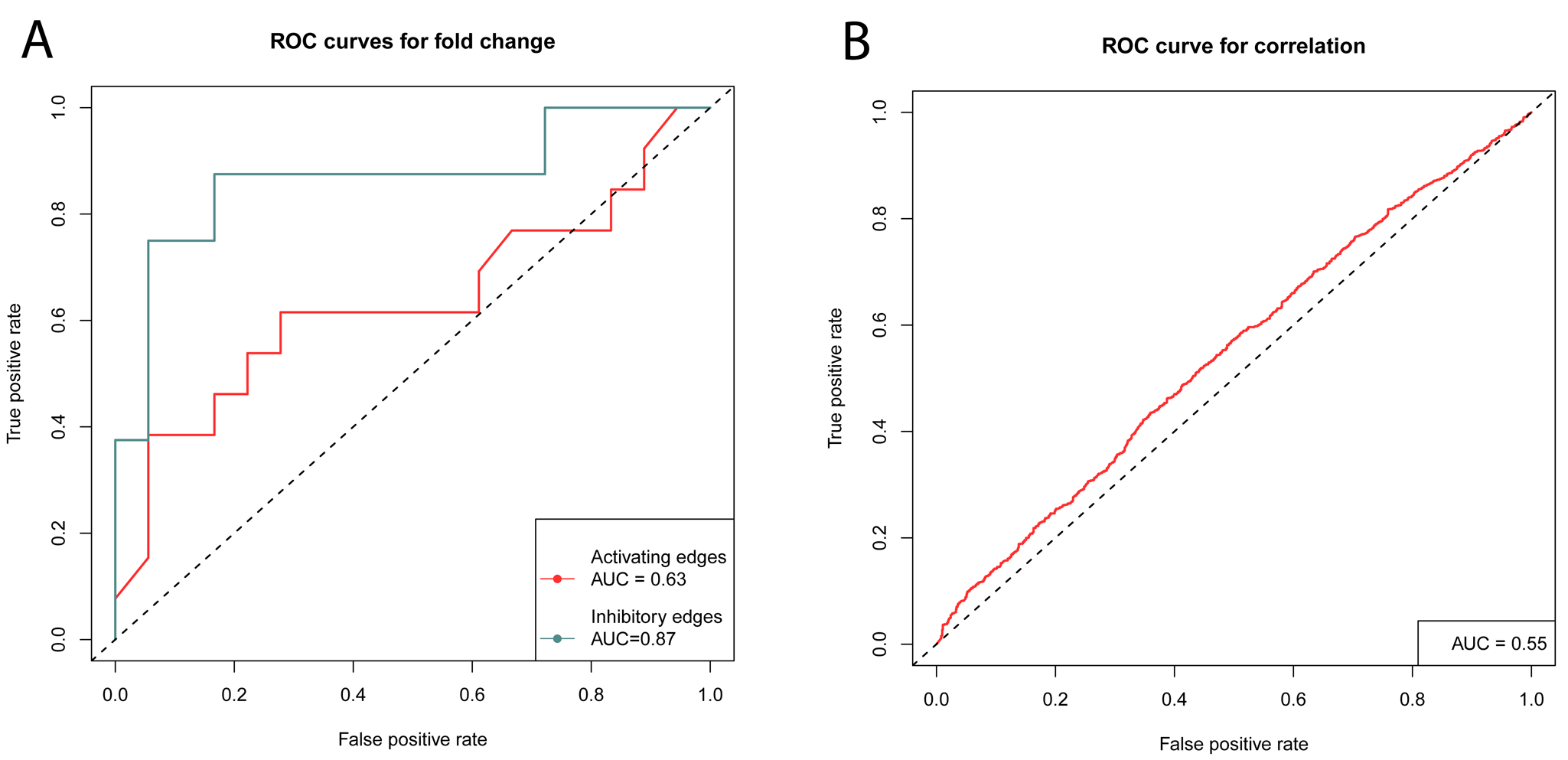

Supplement: Figure S7 — ROC curves showing the ability of perturbation experiments (A) and gene expression correlation (B) to differentiate regulatory from non-regulatory edges. (TIFF) [file pone.0111661.s007.tiff]

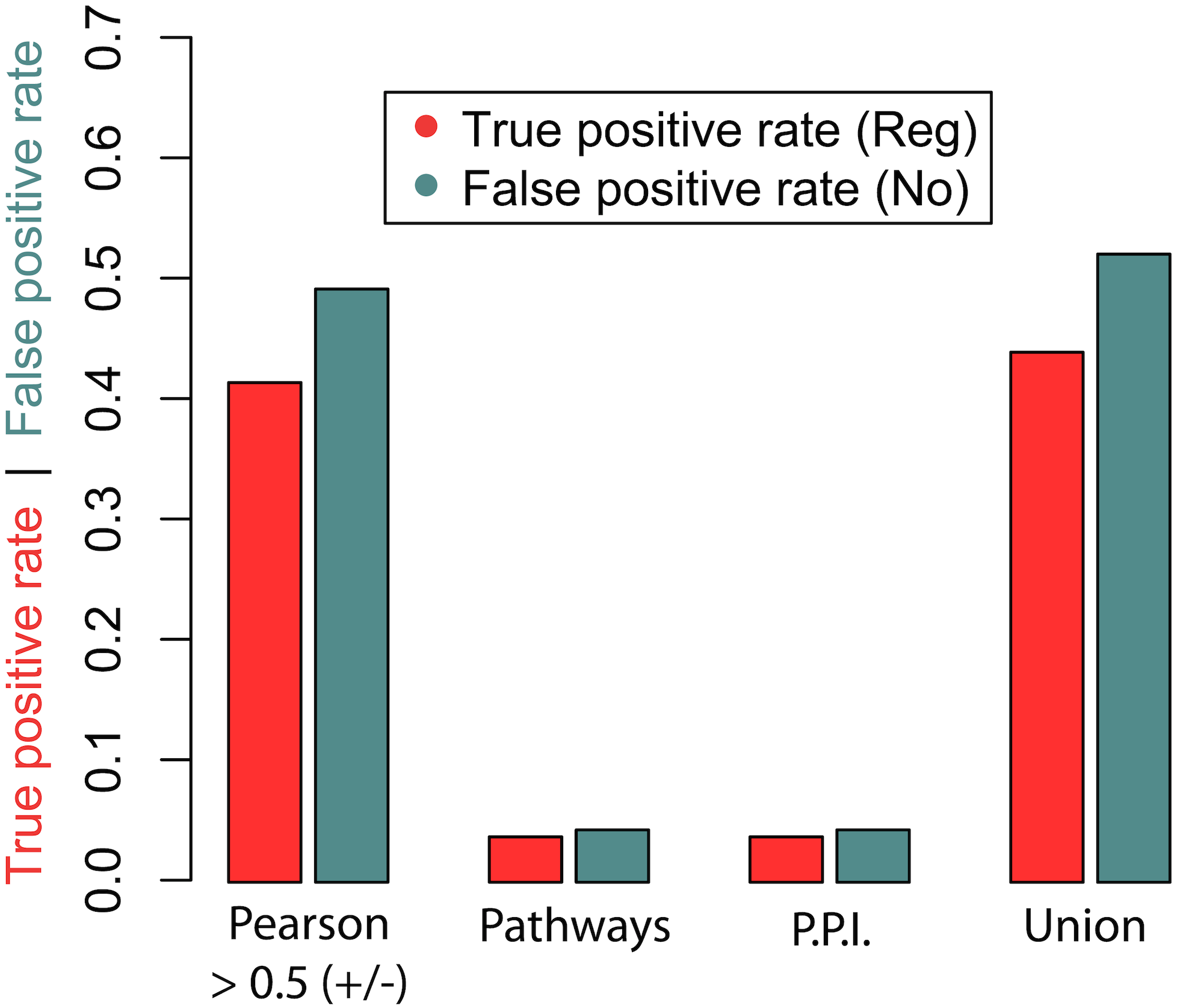

Supplement: Figure S8 — Comparison of the true positive and false positive rates as determined by different network inference approaches on the heart data set: Pearson correlation threshold on 82 microarray profiles, Pathway Commons database, protein-protein interactions (P.P.I.), and the union of the previous three methods. (TIFF) [file pone.0111661.s008.tiff]
