# Supplementary material for: How Difficult Is Inference of Mammalian Causal Gene Regulatory Networks?
Source: PLoS One. 2014 Nov 4;9(11):e111661. doi: 10.1371/journal.pone.0111661 (PMC4219746; doi:10.1371/journal.pone.0111661)
Supplement: Table S1 — Summary of heart microarray dataset. (PDF) [file pone.0111661.s009.pdf]

Table S1. Summary of cardiac microarray data set.

Time series

	GSE1479							GSE11040	
	E10.5	E11.5	E12.5	E13.5	E14.5	E16.5	E18.5	E12.5	E17.5
Whole heart	3	3							
Both ventricle			3	3	3	3	3		
Atrial chamber			3	3	3	3	3		
Endocardial cushions								2	
Atrioventricular valves									2

Genetic perturbation

	GSE28186 IP3R1/IP3R3		GSE41179 Ilk		GSE9124 Sp3		GSE50426 Fog2		GSE6770 Hdac2		GSE45583 Lsd1	
	WT	Mut	WT	Mut	WT	Mut	WT	Mut	WT	Mut	WT	Mut
Whole heart, E9.25	2	2										
Neural crest, E10.5			2	2								
Whole heart, E12.5					2 (/3)	2 (/3)						
Whole heart, E16.5							3	3				
Ventricle, E17.5									2	2		
Whole heart, E18.5											5	3

Phenotypic difference

	GSE32078	
	WT	Diabetic
Whole heart, E13.5	3	3
Whole heart, E15.5	3	3
