# Supplementary material for: How Difficult Is Inference of Mammalian Causal Gene Regulatory Networks?
Source: PLoS One. 2014 Nov 4;9(11):e111661. doi: 10.1371/journal.pone.0111661 (PMC4219746; doi:10.1371/journal.pone.0111661)
Supplement: Table S2 — Summary of the tooth and heart datasets. (PDF) [file pone.0111661.s010.pdf]

Table S2. Summary of tissue and time specific regulatory actions. Full references can be found at ToothCode (http://compbio.med.harvard.edu/ToothCODE/) and CardiacCode (http://cardiacCode.victorchang.edu.au) websites

Heart

Regulator	Target	Tissue	Stage	Mode	Reference
Pax3	Plxna2	Hypaxial muscle	E12.5	act	Brown et al. (2001)
Pax3	Plxna2	Left ventricle	E12.5	inhib	Brown et al. (2001)
Tbx20	Hand2	Right ventricle	E9	act	Takeuchi et al. (2005)
Tbx20	Hand2	Right ventricle, Outflow tract	E9.5	inhib	Singh et al. (2005)
Tcf21	Tcf21	Pharyngeal mesoderm	E9.5	act	Harel and Maezawa (2012)
Tcf21	Tcf21	Pharyngeal mesoderm	E9.75	inhib	Harel and Maezawa (2012)
Wnt3	Lhx1	Mesoderm	E7.5	act	Liu et al. (1999)
Wnt3	Lhx1	Anterior viseral endoderm	E7.5	inhib	Liu et al. (1999)

Tooth

Regulator	Target	Tissue	Stage	Mode	Reference
Bmp4	Dlx2	Epi	E10 and E13	act	Thomas et al. (2000), Liu et al. (2005)
Bmp4	Dlx2	Mes	E11	act	Bei et al. (1998)
Bmp4	Dlx2	Mes	E10	inhib	Thomas et al. (2000), Liu et al. (2005)
Bmp4	Shh	Epi	E11, E12 and E14	act	Fujimotri et al. (2010)
Bmp4	Shh	Epi	E11 and E14	inhib	Zhao et al. (2000)
Ednra	Dlx2	Epi	E10	act	Ruest et al. (2004)
Ednra	Dlx2	Mes	E10	inhib	Ruest et al. (2004)
Fgf8	Dlx2	Mes	E11	act	Thomas et al. (1997), Bei et al. (1998)
Fgf8	Dlx2	Epi	E10	inhib	Thomas et.al (2000)
Sostdc1	Shh	Epi	E14	act	Ohazama et al. (2008)
Sostdc1	Shh	Epi	E13	inhib	Ahn et al. (2010)
